# Hemodynamic effect of pimobendan following intramuscular and intravenous administration in healthy dogs: A pilot study

**DOI:** 10.3389/fvets.2022.969304

**Published:** 2022-10-12

**Authors:** Masayuki Enokizono, Ahmed S. Mandour, Syunta Komeda, Seijirow Goya, Aki Takeuchi, Konosuke Katoh, Akira Yairo, Zeki Yilmaz, Kazumi Shimada, Ryou Tanaka

**Affiliations:** ^1^Department of Veterinary Surgery, Faculty of Veterinary Medicine, Tokyo University of Agriculture and Technology, Tokyo, Japan; ^2^Department of Animal Medicine (Internal Medicine), Faculty of Veterinary Medicine, Suez Canal University, Ismailia, Egypt; ^3^Department of Bioresource Sciences, Nihon University, Fujisawa, Japan; ^4^Department of Internal Medicine, Faculty of Veterinary Medicine, Uludag University, Bursa, Turkey

**Keywords:** pimobendan injection, echocardiography, hemodynamics, phosphodiesterase III inhibitor, cardiotonic, dogs

## Abstract

**Background:**

Pimobendan is widely used for the treatment of dogs with heart failure *via* the oral route. A new injectable form of pimobendan is now available and its potential usefulness *via* intravenous route has been recently demonstrated in dogs. However, the cardiovascular effects of intramuscular (IM) administration of injectable pimobendan have not been investigated yet.

**Hypothesis:**

IM administration of pimobendan may have the same hemodynamic effect as the IV route.

**Methods:**

Six healthy Beagle dogs underwent a placebo-controlled double-blind crossover study. The early cardiovascular effects after a single dose of IM and IV injections of pimobendan (0.2 ml/kg; Pimo IM and Pimo IV, respectively) were compared to the same volume of IM placebo (Saline IM) in anesthetized dogs. Clinical [heart rate (HR) and blood pressure (BP)] and echocardiographic hemodynamic parameters [left ventricular (LV) inflow waveforms of diastolic early wave (eV), atrial systolic wave (aV), diastolic early mitral ring velocity (e′), peak velocity (pV), stroke volume (SV), cardiac output (CO), and systemic vascular resistance (SVR)] were monitored with 15 min intervals for 120 min.

**Results:**

Diastolic BP decreased significantly at 30 min in Pimo IM compared to Saline IM. Mean eV and CO values significantly increased from 75 min, e′ from 60 min, pV from 75 min, and SV from 15 to 120 min, whereas SVR significantly decreased at 30–60 min in Pimo IM compared to those of Saline IM (*P* < 0.05). Compared with the Pimo IV, eV and pV were significantly lower at 30–60 min (*P* < 0.05) while SV was significantly higher at 90–105 min in Pimo IM (*P* < 0.05). Other hemodynamic parameters (BP, HR, SVR, CO, e′, and E/e′) did not significantly change between Pimo IM and IV.

**Conclusions:**

The hemodynamic effect of pimobendan following IM and IV injection was described. Our results suggested that IM administration of pimobendan is equally comparable and possibly interchangeable with IV administration. This warrant further studies to investigate the clinical effectiveness of IM pimobendan in treating dogs with congestive heart failure or in heart failure cases unable to receive IV or oral administration.

## Introduction

Mitral valve disease (MVD) is the most common acquired heart disease in dogs which accounts for 75–80% of congestive heart failure (1, 2). Pimobendan has long been used as a treatment of choice in dogs suffering from MVD (3–5) and dilated cardiomyopathy (DCM) (6). ACVIM Specialists of Cardiology recommended the use of pimobendan for hospital-based acute care to improve the survival rate and patient outcomes (7, 8). Moreover, pimobendan medication delays the onset of congestive heart failure (CHF) in dogs with preclinical MVD with enlarged left atrium and left ventricle (ACVIM Stage B2) by 15 months (3). Pimobendan has cardiotonic, myocardial relaxing, and vasodilatory effects by calcium sensitivity enhancing (9–11) and phosphodiesterase III (PDE-III) inhibitory effects (12–14) in a dose-dependent manner (15).

Oral administration of pimobendan is the most preferred method when the MVD condition is stable and for chronic medication strategy (16). The pharmacokinetics of pimobendan during oral medication considering the drug form vary among studies. This difference is essential to predict the onset of the peak effect of pimobendan during the treatment of patients, particularly in severe cases (17–20). In addition, many dogs with acute heart failure cases due to MDV have little reserve cardiorespiratory function, hemoptysis, and respiratory distress (2). Oral administration in such cases is more difficult than parenteral routes, and the desired effect of the drug occurs slowly when used per os (17), making it an inappropriate route of administration in emergency cases such as in acute heart failure or severe CHF.

Currently, injectable pimobendan has been formulated and became available for IV injection (17, 21), suggesting an alternative live-saving route of administration in case of advanced stages of MVD to rapidly reduce the left ventricular end-diastolic pressure (17, 22). Recent studies revealed that the IV administration of pimobendan, but not SC, may be useful in patients with acute heart failure caused by MVD (23, 24). Until now, there is no data regarding the IM route of pimobendan administration in dogs. In severe cases, injection of medicine is a lifesaving choice because of getting the desired effect after a short time compared with the oral route. In light of previous literature, there are certain cases of emergency where the IV route can be accessed easily. However, in some cases, like vasogenic shock, IV is very difficult due to the collapse of peripheral veins (25). Also, in small breed dogs, the peripheral veins are not so easily accessed and IV injection should perform with caution. When we consider these points, the IM route of pimobendan administration is worth studying. Practically, IM injection is easier, and safer, and may serve as an alternative route of administration in urgent cases when the IV route is difficult. Besides, the desired effect of the medication is generally more rapid when used *via* IM than the SC (26, 27).

The current study aims to investigate the cardiovascular effect of injectable pimobendan through the IM route. We will demonstrate the prolonged effect of pimobendan IM injection on cardiovascular functions through evaluation of hemodynamic changes, evaluating the start time of action, the duration for maximum activity, and the strength of the action in comparison with the IV route in healthy dogs.

## Materials and methods

### Animals

A group of six female beagle dogs aged 1–3 years old and weighing 7.6–8.4 kg (median: 7.9 kg) was studied. No abnormalities were found in their health, based on clinical, hematological, and serum biochemical examinations, as well as the cardiologic evaluation by electrocardiography and transthoracic echocardiography.

### Study design

This is a placebo-controlled, double-blinded, crossover study. In this study, the cardiovascular effect of pimobendan treatment *via* IM route (Pimo IM) was investigated which was compared with pimobendan IV (Pimo IV) and placebo administered IM (normal saline; saline IM).

For each examined dog, the supervisors randomly prepared the dose of injectable pimobendan and the same volume of saline as Drugs A and B. Additionally, the other supervisor selected either Drug A or B for IM administration and subsequent measurements of heart function parameters in anesthetized animals. The IM injections were performed in the longissimus muscle of the lower back. None of the supervisors who prepared and administered the drug/placebo knew which substance was administered until all experiments were finished. In addition, the preparation and administration of the drug were overseen by the designated supervisors. Moreover, the effect of intravenous administration of pimobendan (Pimo IV) was compared with that of the Pimo IM, using the same individual animals as in the Pimo IM vs. saline IM test. The drugs were prepared at doses of pimobendan 0.15 mg/kg (0.2 ml/kg; Vetmedin^®^ 0.75 mg/ml, Boehringer Ingelheim, Germany) for IM and IV routes, and saline 0.2 ml/kg (Otsuka normal saline, Otsuka Pharmaceutical Factory, Inc., Japan), respectively, according to the previously published protocol (23). The IV route was injected over 1 min in the cephalic vein.

In this experiment, all dogs were subjected to the same route of administration. After one week's lag, another route was started to avoid overlapping and ensure complete clearance of the medicine (23).

### Anesthesia and preparation

A catheter (SURFLO 22-gauge, 25 mm, TERUMO, Japan) was placed in the cephalic vein for pre-medication and infusion. Each dog was pre-medicated with butorphanol (0.2 mg/kg, IV; Vetorphale, Meiji Seika, Japan). Anesthesia was slowly introduced with propofol IV (6.0 mg/kg; propofol IV 1% “FK”, Fresenius Kabi Japan K.K., Japan) and endotracheal intubation was performed (28, 29). General anesthesia was maintained by isoflurane (isoflurane, Pfizer Inc., NY, United States) to 100% oxygen, supplied *via* mechanical ventilation (Aestiva/5, Datex-Ohmeda, GE Healthcare, Japan). The concentration of isoflurane during the experiment was determined for each dog (1.0–1.8%), and the concentration was maintained within a range of ±0.1%. The dog was placed in the left lateral position, and the heart rate (HR), expiratory terminal carbon dioxide partial pressure (PaCO_2_), expiratory terminal isoflurane concentration, blood pressure [BP; systolic blood pressure (SBP), mean blood pressure (MAP), diastolic blood pressure (DBP)], esophageal temperature, and saturation of percutaneous oxygen (SpO_2_) were continuously monitored using an anesthesia monitor (Life Scope A BSM-5192, Nihon Kohden, Japan). Samples for expiratory terminal PaCO_2_ and expiratory terminal isoflurane concentration were collected from the Y piece of the ventilator respiratory system, and expiratory terminal PaCO_2_ was maintained at 35–45 mmHg. BP was monitored with a transducer (DX-300, Nihon Kohden, Japan) connected with a catheter (SURFLO 24-gauge, 19 mm, TERUMO, Japan) placed in the dorsalis pedis artery. Esophageal temperature was maintained at 37.0–37.5°C using a warm air mat (Bair hugger™, 3M™, Minnesota, United States). The SpO_2_ was measured with a pulse oximeter clipped to the tongue and levels were maintained at 98% or higher. Lactated Ringer's solution was infused at a flow rate of 3.0 mL/kg/h throughout the experiment.

### Measurements of cardiovascular and hemodynamic parameters

After confirming the stability of the hemodynamic parameters following anesthesia, the baseline parameters before drug administration (Time 0) were measured from the anesthesia monitor and by using conventional echocardiography (Prosound F75, Hitachi Aloka Med., Japan). Subsequently, while dogs were still under anesthesia, drug administration was started, and the data were measured every 15 min up to 120 min (eight-time points: T15, T30, T45, T60, T75, T90, T105, T120) after the acquisition of the baseline measurements (T0). This protocol has been modified from previously published studies (17, 23). For echocardiography (30), a cross-sectional image of the apex of the heart was visualized. By the left parasternal approach, the left ventricular inflow waveforms of the diastolic early wave (eV) and atrial systolic wave (aV) were measured using the pulse Doppler method, and the early diastolic mitral annular velocity (assessed by TDI) (e′) on the lateral wall was measured using the tissue Doppler echocardiography. The eV/e′ (E/e′) was calculated from the measured eV and e′. Subsequently, the five-chamber view with color flow Doppler was acquired, and the peak velocity (pV), velocity-time integral (VTI), and tract diameter (d, cm) of LV outflow was measured by the Pulsed-wave Doppler echocardiography of the aorta. The following values were calculated from the following formulas (31):

Stroke volume (SV) = (d/2)^2^ × π × VTI
Cardiac output (CO) = SV × HR
Systemic vascular resistance (SVR) = 80.0 × (MAP – central venous pressure).

Since the healthy dogs had no right atrial enlargement or transvenous distension, the central venous pressure was assumed to be 5 mmHg (32).

### Statistical analysis

The statistical analysis was performed by SATISTA (Kyoto, Japan). SPSS for Windows version 24.0 (IBM Japan, Tokyo, Japan) was used for the statistical analysis. For each time series outcome, the time (every 15 min), group (saline IM, Pimo IM, Pimo IV), and their interactions were fixed effects; additionally, RM-ANCOVA was performed with the initial value of the dependent variable (Time 0) corrected as a covariant. The estimated mean value at each time and its 95% confidence interval (CI) were calculated. For the pairwise comparison, the Pimo IM vs. saline IM test and the Pimo IV vs. IM test were performed. Since the comparative tests of the above two hypotheses were performed independently of each other, multiplicity was not corrected. Since each outcome was researched on the assumption that it followed a normal distribution from the preliminary survey, a parametric method was adopted. *P* < 0.05 was considered statistically significant.

## Results

### Hemodynamic changes caused by pimobendan IM in comparison with the baseline

All hemodynamic measurements obtained from the current study are submitted as additional data (Supplementary Tables 1, 2). Tables 1, 2 summarizes the changes in hemodynamic parameters after pimobendan administration in comparison with the baseline (Time 0). The HR was significantly higher than the baseline 30 and 90 min after administration of Pimo IM. The SBP and MAP did not change significantly during the monitoring period (*P* > 0.05); however, DBP showed a significant decrease at 60, 75, and 90 min compared to its baseline (*P*<*0.05*, Table 1).

**Table 1:** Heart rate and blood pressure measurements after pimobendan injection in comparison with measurements at the baseline (Time 0).

**Time (min)**	**Saline IM**				**Pimobendan IM**				**Pimobendan IV**			
	**Mean**	**95%CI**		***P*-value**	**Mean**	**95%CI**		***P*-value**	**Mean**	**95%CI**		***P*-value**
**HR (beats/min)**												
15	2.14	−1.09	5.37	0.18	0.20	−3.04	3.43	0.90	2.50	−0.73	5.72	0.12
30	4.04	0.97	7.11	**0.01**	3.79	0.72	6.86	**0.02**	3.17	0.11	6.24	**0.04**
45	3.62	0.00	7.23	**0.05**	3.40	−0.22	7.01	0.06	1.66	−1.95	5.26	0.34
60	6.50	1.70	11.30	**0.01**	3.90	−0.90	8.70	0.10	−0.40	−5.19	4.39	0.86
75	6.32	−0.31	12.96	0.06	−0.76	−7.39	5.88	0.81	4.60	−2.02	11.22	0.16
90	8.26	3.45	13.07	**0.002**	5.96	1.14	10.77	**0.02**	3.79	−1.01	8.59	0.11
105	8.78	2.69	14.88	**0.01**	9.00	2.90	15.10	**0.01**	6.55	0.47	12.64	**0.04**
120	10.31	2.83	17.80	**0.01**	10.83	3.34	18.32	**0.01**	6.86	−0.61	14.33	0.07
**SBP (mmHg)**												
15	−2.10	−8.26	4.06	0.48	−2.02	−8.43	4.39	0.51	−0.88	−7.32	5.56	0.78
30	0.58	−4.15	5.32	0.80	−2.90	−7.82	2.03	0.23	−4.35	−9.30	0.59	0.08
45	−3.85	−9.39	1.70	0.16	−4.85	−10.62	0.92	0.09	−1.30	−7.10	4.50	0.64
60	−3.82	−11.67	4.03	0.31	−3.81	−11.97	4.35	0.33	0.46	−7.74	8.67	0.91
75	−3.16	−11.25	4.93	0.42	−4.03	−12.44	4.38	0.32	−1.31	−9.76	7.15	0.75
90	0.00	−7.36	7.37	1.00	−6.13	−13.79	1.53	0.11	−2.71	−10.40	4.99	0.46
105	−0.97	−9.64	7.70	0.81	−2.74	−11.75	6.28	0.53	−0.62	−9.69	8.44	0.89
120	0.86	−7.17	8.89	0.82	−3.79	−12.13	4.56	0.35	0.76	−7.63	9.15	0.85
**MAP (mmHg)**												
15	0.93	−2.37	4.23	0.55	0.25	−3.06	3.57	0.87	0.32	−3.11	3.74	0.85
30	3.08	−0.28	6.43	0.07	−0.45	−3.82	2.92	0.78	−1.30	−4.78	2.19	0.44
45	2.19	−1.19	5.56	0.19	−0.59	−3.99	2.80	0.71	−1.26	−4.77	2.25	0.45
60	1.64	−2.78	6.06	0.44	−1.18	−5.62	3.26	0.58	−0.46	−5.05	4.13	0.83
75	1.99	−1.88	5.86	0.29	−1.96	−5.86	1.93	0.30	−0.52	−4.54	3.50	0.78
90	2.64	−1.31	6.59	0.17	−2.31	−6.28	1.66	0.23	−0.99	−5.10	3.11	0.61
105	2.31	−2.48	7.10	0.32	0.15	−4.67	4.97	0.95	−0.62	−5.60	4.35	0.79
120	3.23	−1.08	7.54	0.13	−0.78	−5.12	3.55	0.70	0.56	−3.92	5.03	0.79
**DBP (mmHg)**												
15	0.69	−3.47	4.85	0.73	−2.17	−6.19	1.86	0.27	1.47	−2.68	5.63	0.46
30	3.54	−0.90	7.99	0.11	−3.00	−7.30	1.30	0.16	−0.21	−4.65	4.23	0.92
45	3.02	−0.91	6.94	0.12	−2.17	−5.96	1.63	0.24	−1.85	−5.78	2.07	0.33
60	1.51	−2.23	5.25	0.40	−3.67	−7.28	−0.05	**0.05**	−1.18	−4.92	2.56	0.51
75	1.57	−1.93	5.08	0.35	−4.33	−7.72	−0.94	**0.02**	−1.57	−5.08	1.93	0.35
90	2.72	−1.25	6.69	0.16	−4.67	−8.50	−0.83	**0.02**	−1.39	−5.35	2.58	0.47
105	1.56	−2.48	5.60	0.42	−1.00	−4.91	2.91	0.59	−1.56	−5.60	2.48	0.42
120	1.64	−2.20	5.48	0.37	−2.17	−5.88	1.55	0.23	−0.48	−4.32	3.36	0.79

Test for change from Time 0 in the repeated measures ANCOVA (time effect, group effect, and time^*^group interaction) (least significant difference). P-value of 0.05 is considered significant. 95% CI: 95% confidence interval. HR, heart rate; SBP, systolic blood pressure; DBP, diastolic blood pressure; MBP, mean blood pressure. Bold values idicated that the P value < 0.05.

**Table 2:** Hemodynamic parameters after pimobendan injection in comparison with the baseline (Time 0).

**Time**	**Saline IM**				**Pimobendan IM**				**Pimobendan IV**			
	**Mean**	**95%CI**		***P*-value**	**Mean**	**95%CI**		***P*-value**	**Mean**	**95%CI**		***P*-value**
**aV (cm/s)**												
15	1.71	−2.83	6.25	0.43	3.24	−1.50	7.98	0.17	4.34	−0.40	9.07	0.07
30	3.03	−1.36	7.42	0.16	2.13	−2.45	6.71	0.34	6.39	1.82	10.97	**0.01**
45	2.47	−1.80	6.75	0.24	4.75	0.29	9.21	**0.04**	8.36	3.90	12.81	**0.001**
60	2.89	−2.33	8.11	0.26	6.93	1.48	12.38	**0.02**	7.23	1.78	12.67	**0.01**
75	3.79	−1.87	9.44	0.17	7.66	1.76	13.57	**0.02**	7.89	2.00	13.79	**0.01**
90	4.59	0.40	8.78	**0.03**	9.03	4.66	13.41	**< 0.001**	11.73	7.36	16.10	**< 0.001**
105	4.43	−1.72	10.58	0.15	12.44	6.02	18.86	**< 0.001**	15.18	8.77	21.60	**< 0.001**
120	7.55	2.67	12.42	**0.01**	13.18	8.10	18.27	**< 0.001**	12.07	6.99	17.15	**< 0.001**
**eV (cm/s)**												
15	2.84	−3.04	8.72	0.32	7.23	1.28	13.17	**0.02**	12.35	7.07	17.62	**< 0.001**
30	3.33	−5.92	12.59	0.45	7.35	−2.01	16.71	0.11	20.33	12.03	28.63	**< 0.001**
45	3.03	−9.07	15.14	0.60	12.39	0.14	24.63	**0.05**	26.09	15.23	36.94	**< 0.001**
60	6.65	−5.17	18.47	0.25	19.20	7.25	31.16	**0.004**	31.05	20.45	41.66	**< 0.001**
75	3.60	−5.14	12.33	0.39	34.89	26.05	43.73	**< 0.001**	33.51	25.68	41.35	**< 0.001**
90	6.04	−5.79	17.86	0.29	23.87	11.91	35.83	**< 0.001**	31.22	20.61	41.82	**< 0.001**
105	7.51	−4.19	19.22	0.19	35.28	23.44	47.11	**< 0.001**	34.88	24.38	45.38	**< 0.001**
120	4.75	−4.34	13.83	0.28	28.63	19.44	37.81	**< 0.001**	35.61	27.46	43.75	**< 0.001**
**e** **′** **(cm/s)**												
15	0.07	−0.36	0.50	0.74	0.41	−0.03	0.84	0.06	0.75	0.31	1.18	**0.002**
30	0.41	−0.58	1.39	0.39	0.64	−0.35	1.64	0.19	1.94	0.94	2.93	**0.001**
45	0.16	−0.94	1.26	0.76	0.76	−0.35	1.87	0.16	2.14	1.03	3.25	**0.001**
60	−0.04	−1.18	1.10	0.94	1.85	0.70	3.00	**0.004**	2.85	1.71	4.00	**< 0.001**
75	−0.04	−1.48	1.40	0.95	1.68	0.23	3.13	**0.03**	2.96	1.51	4.41	**0.001**
90	0.51	−0.61	1.62	0.34	2.59	1.47	3.71	**< 0.001**	2.95	1.83	4.07	**< 0.001**
105	0.06	−1.26	1.37	0.93	2.13	0.81	3.45	**0.004**	3.28	1.96	4.60	**< 0.001**
120	0.01	−1.18	1.20	0.99	2.51	1.31	3.71	**0.001**	2.78	1.58	3.99	**< 0.001**
**E/e** **′**												
15	0.7	0.0	1.4	**0.04**	0.15	−0.51	0.81	0.63	0.75	0.10	1.40	**0.03**
30	0.3	−1.0	1.5	0.66	0.08	−1.10	1.27	0.88	0.74	−0.43	1.92	0.19
45	0.5	−0.5	1.4	0.29	0.45	−0.48	1.37	0.32	1.15	0.24	2.06	**0.02**
60	1.2	−0.1	2.4	0.06	0.00	−1.18	1.18	0.99	1.08	−0.08	2.25	0.07
75	1.0	−0.2	2.3	0.11	1.51	0.27	2.75	**0.02**	1.31	0.09	2.54	**0.04**
90	0.5	−0.4	1.4	0.24	0.00	−0.85	0.85	0.99	0.85	0.01	1.69	**0.05**
105	1.1	−0.1	2.3	0.07	1.42	0.23	2.61	**0.02**	0.94	−0.23	2.12	0.11
120	0.6	−0.2	1.5	0.13	0.71	−0.12	1.53	0.09	1.23	0.41	2.04	**0.01**
**pV (cm/s)**												
15	7.95	2.45	13.44	**0.01**	9.95	4.55	15.34	**0.001**	13.49	8.17	18.81	**< 0.001**
30	12.94	4.47	21.41	**0.01**	24.88	16.56	33.20	**< 0.001**	28.26	20.06	36.46	**< 0.001**
45	16.05	5.88	26.21	**0.004**	28.62	18.63	38.61	**< 0.001**	48.58	38.74	58.42	**< 0.001**
60	11.61	−5.20	28.42	0.16	33.02	16.50	49.53	**< 0.001**	60.08	43.81	76.35	**< 0.001**
75	15.00	0.54	29.45	**0.04**	60.45	46.25	74.65	**< 0.001**	63.69	49.70	77.68	**< 0.001**
90	15.46	2.05	28.87	**0.03**	70.63	57.45	83.80	**< 0.001**	55.21	42.23	68.19	**< 0.001**
105	22.72	6.71	38.72	**0.01**	68.68	52.95	84.40	**< 0.001**	55.25	39.76	70.74	**< 0.001**
120	26.72	16.94	36.50	**< 0.001**	59.90	50.29	69.51	**< 0.001**	63.88	54.41	73.35	**< 0.001**
**SV (mL)**												
15	0.46	−0.73	1.65	0.42	2.24	1.00	3.47	**0.002**	1.58	0.39	2.76	**0.01**
30	1.19	−0.43	2.81	0.14	3.86	2.18	5.53	**< 0.001**	4.27	2.65	5.88	**< 0.001**
45	2.22	0.60	3.83	**0.01**	4.69	3.01	6.37	**< 0.001**	6.24	4.63	7.86	**< 0.001**
60	1.28	−1.14	3.69	0.28	5.73	3.22	8.24	**< 0.001**	7.70	5.28	10.11	**< 0.001**
75	2.32	−0.35	4.99	0.08	9.37	6.60	12.15	**< 0.001**	7.12	4.45	9.79	**< 0.001**
90	1.96	−0.11	4.02	0.06	11.69	9.55	13.84	**< 0.001**	6.63	4.56	8.69	**< 0.001**
105	2.57	1.26	3.88	**< 0.001**	8.73	7.37	10.10	**< 0.001**	6.27	4.96	7.58	**< 0.001**
120	2.26	0.31	4.20	**0.03**	9.33	7.31	11.35	**< 0.001**	6.95	5.00	8.89	**< 0.001**
**CO (L/min)**												
15	0.16	0.01	0.32	**0.04**	0.22	0.05	0.39	**0.01**	0.24	0.08	0.40	**0.01**
30	0.17	−0.05	0.40	0.12	0.49	0.24	0.73	**< 0.001**	0.50	0.27	0.73	**< 0.001**
45	0.32	−0.17	0.80	0.18	0.83	0.31	1.34	**0.004**	0.67	0.18	1.16	**0.01**
60	0.24	−0.07	0.56	0.12	0.62	0.29	0.96	**0.001**	0.76	0.44	1.08	**< 0.001**
75	0.34	−0.07	0.75	0.10	1.09	0.65	1.53	**< 0.001**	0.82	0.40	1.23	**< 0.001**
90	0.39	0.06	0.72	**0.02**	1.21	0.86	1.56	**< 0.001**	0.90	0.57	1.23	**< 0.001**
105	0.47	0.18	0.76	**0.004**	1.03	0.72	1.33	**< 0.001**	0.90	0.60	1.19	**< 0.001**
120	0.42	0.07	0.77	**0.02**	1.15	0.77	1.52	**< 0.001**	0.92	0.56	1.28	**< 0.001**
**SVR (dyne·s/cm** ^**−5**^ **)**												
15	−180.5	−472.2	111.2	0.21	−393.7	−714.3	−73.0	**0.02**	−512.5	−818.2	−206.8	**0.003**
30	−168.6	−432.6	95.3	0.19	−682.8	−972.9	−392.7	**< 0.001**	−985.0	−1261.6	−708.4	**< 0.001**
45	−414.9	−726.0	−103.9	**0.01**	−833.7	−1175.7	−491.8	**< 0.001**	−1283.7	−1609.8	−957.7	**< 0.001**
60	−337.8	−657.0	−18.7	**0.04**	−853.6	−1204.4	−502.7	**< 0.001**	−1315.7	−1650.2	−981.1	**< 0.001**
75	−442.4	−891.6	6.8	0.05	−1270.4	−1764.2	−776.6	**< 0.001**	−1306.4	−1777.2	−835.6	**< 0.001**
90	−427.1	−749.0	−105.2	**0.01**	−1404.5	−1758.4	−1050.7	**< 0.001**	−1368.9	−1706.3	−1031.5	**< 0.001**
105	−483.0	−853.8	−112.2	**0.01**	−1193.0	−1600.6	−785.4	**< 0.001**	−1353.8	−1742.4	−965.1	**< 0.001**
120	−537.4	−774.6	−300.2	**< 0.001**	−1300.2	−1561.0	−1039.4	**< 0.001**	−1358.9	−1607.6	−1110.3	**< 0.001**

Test for change from Time 0 in the repeated measures ANCOVA (time effect, group effect, and time^*^group interaction) (least significant difference). P-value of 0.05 is considered significant. 95% CI: 95% confidence interval.

aV, atrial systolic wave; eV, the early diastolic wave; e′, the diastolic early mitral ring velocity; The eV/e′ (E/e′) early mitral inflow to diastolic annular tissue velocity; pV, the peak aortic velocity; SV, stroke volume; Co, cardiac output; SVR, systemic vascular resistance. The bold values indicate the values which are less than 0.05.

The echocardiographic data showed significant increases in pV, SV, and CO following Pimo IM at all measurement time points compared with the baseline (Time 0) and reach the maximum values at 90 min (Table 2). The pV was increased from the baseline (mean: 81.4 ± 5.4 cm/s) to (mean: 106.2 ± 8.3 cm/s) after 15 min and continued to increase until 90 min (mean: 152.0 ± 13.1 cm/m). Concomitantly, SV and CO were elevated from the baseline (mean: 13.7 ± 1.2 mL, 1.3 ± 0.1 L/min) to (mean: 15.9 ± 1.2 mL, 1.5 ± 0.1 L/min) after 15 min and reach their maximum values at 90 min (mean: 25.4 ± 2.1 mL, 2.5 ± 0.3 L/min), respectively. The eV increased significantly at 15 min and 45 min after IM administration and reached the maximum value at 105 min after administration. Meanwhile, a significant increase in aV was observed from 45 min after administration, and the increase continued until 120 min. Subsequently, e′ was significantly increased 60 min after administration, and reached a maximum value 90 min after administration. E/e′ index was significantly increased 75 min and 105 min after administration.

Finally, SVR showed a regular manner of a significant decrease in overall measurement time points, showing a minimum reduction at 90 min (mean: 1693 ± 353 dyne·s/cm^5^) after administration compared with the baseline (mean: 3098.4 dyne·s/cm^−5^).

### Hemodynamic changes caused by IV administration of pimobendan in comparison with the baseline

The HR was significantly higher than the baseline at 30 and 105 min after administration. No significant changes were observed in the SBP, MAP, and DBP compared to the baseline. There was a significant increase in the pV, SV, and CO at all measurement points compared with the baseline. The data revealed increased pV from baseline (81.41 ± 5.32 cm/s) and reach its maximum at 75 min (145.10 ± 13.99 cm/s). Besides, the LVOT revealed a continuous increment of SV and CO from Time 0 (13.73 ± 1.19 mL, 1.3 ± 0.16 L/min; respectively) until 60 min for SV (21.43 ± 2.42 mL); while CO continued to increase until 90 min (2.20 ± 0.33 L/min).

While eV and e′ were significantly increased at all measurement points, E/e′ was significantly increased at five measurement points (15, 45, 75, 90, and 120 min) and aV was only significantly increased after 30 min of IV administration.

Furthermore, SVR was significantly reduced at all the measurement points following IV administration of pimobendan and the reduction was continuously observed from the baseline until 90 min (3098.4± 305.7 vs. 1729.5 ± 337.4 dyne·s/cm^−5^).

### Comparison between pimobendan IM and saline IM

**Figure 1:**
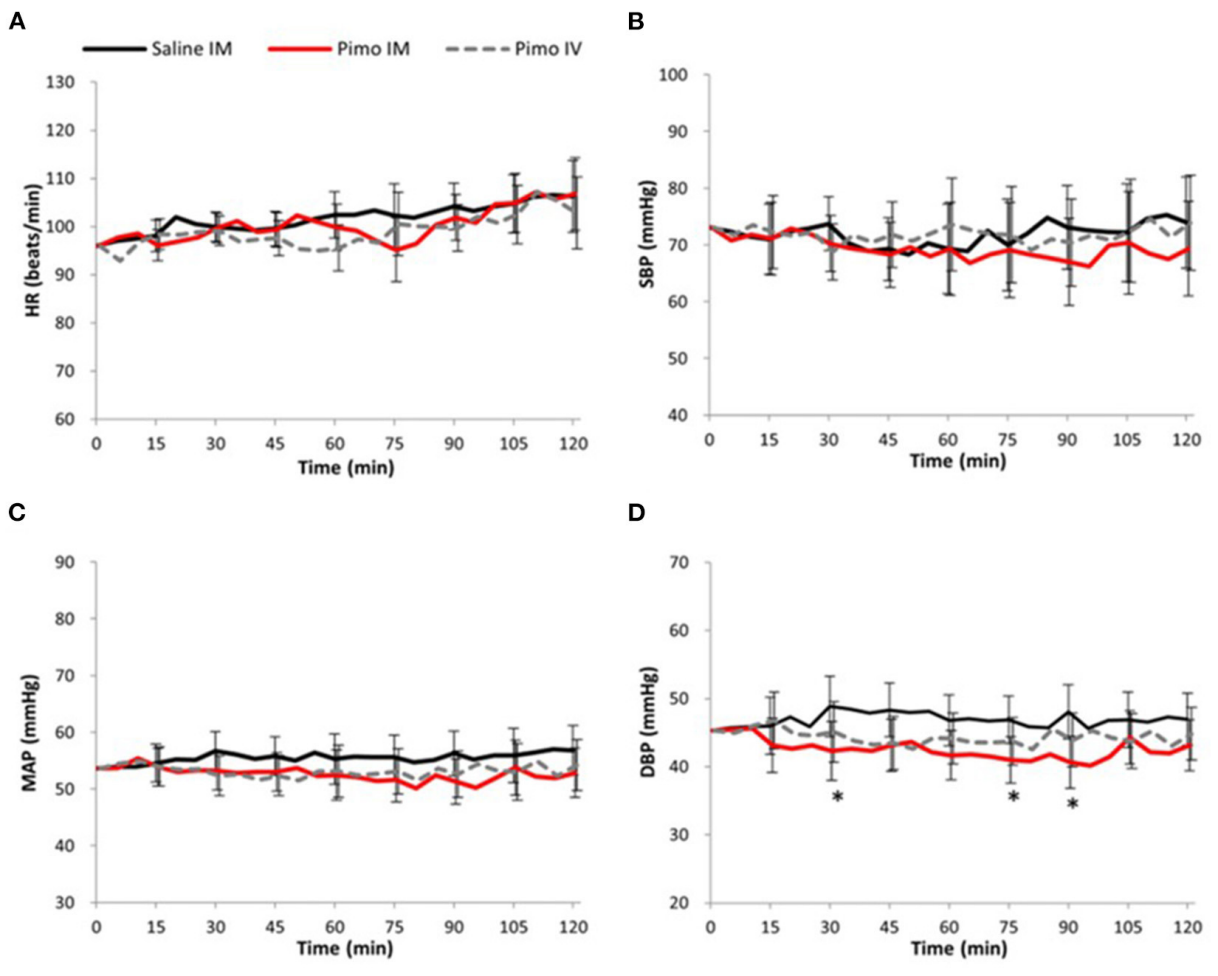
Data are presented as mean ± 95% confidence interval (error bar). Black line: Saline IM; Red line: Pimobendan IM (Pimo IM); Gray dot line: Pimobendan IV (Pimo IV). (*) fitted to indicate the significance difference between Pimo IM Saline IM, [for the repeated measures ANCOVA (model: time, group, and time*group)]; *P* < 0.05. **(A)** Heat rate (HR, beat/min), **(B)** systolic blood pressure (SBP, mmHg), **(C)** mean arterial pressure (MAP, mmHg), **(D)** diastolic blood pressure (DBP, mmHg).

No significant difference between groups was observed at all measurement points in HR, SBP, or MAP (Figures 1A–C). However, a significant decrease was observed in DBP in Pimo IM at 30 min [difference between groups (“Pimo IM,” “Saline IM”): −6.54 (95% CI: −12.72 - −0.36), *P* = 0.040], 75 min [−5.91 (CI: −10.78 - −1.03), *P* = 0.021], and 90 min [−7.39 (CI: −12.91 - −1.87), *P* = 0.012] after administration compared with those of Saline IM (Figure 1D).

**Figure 2:**
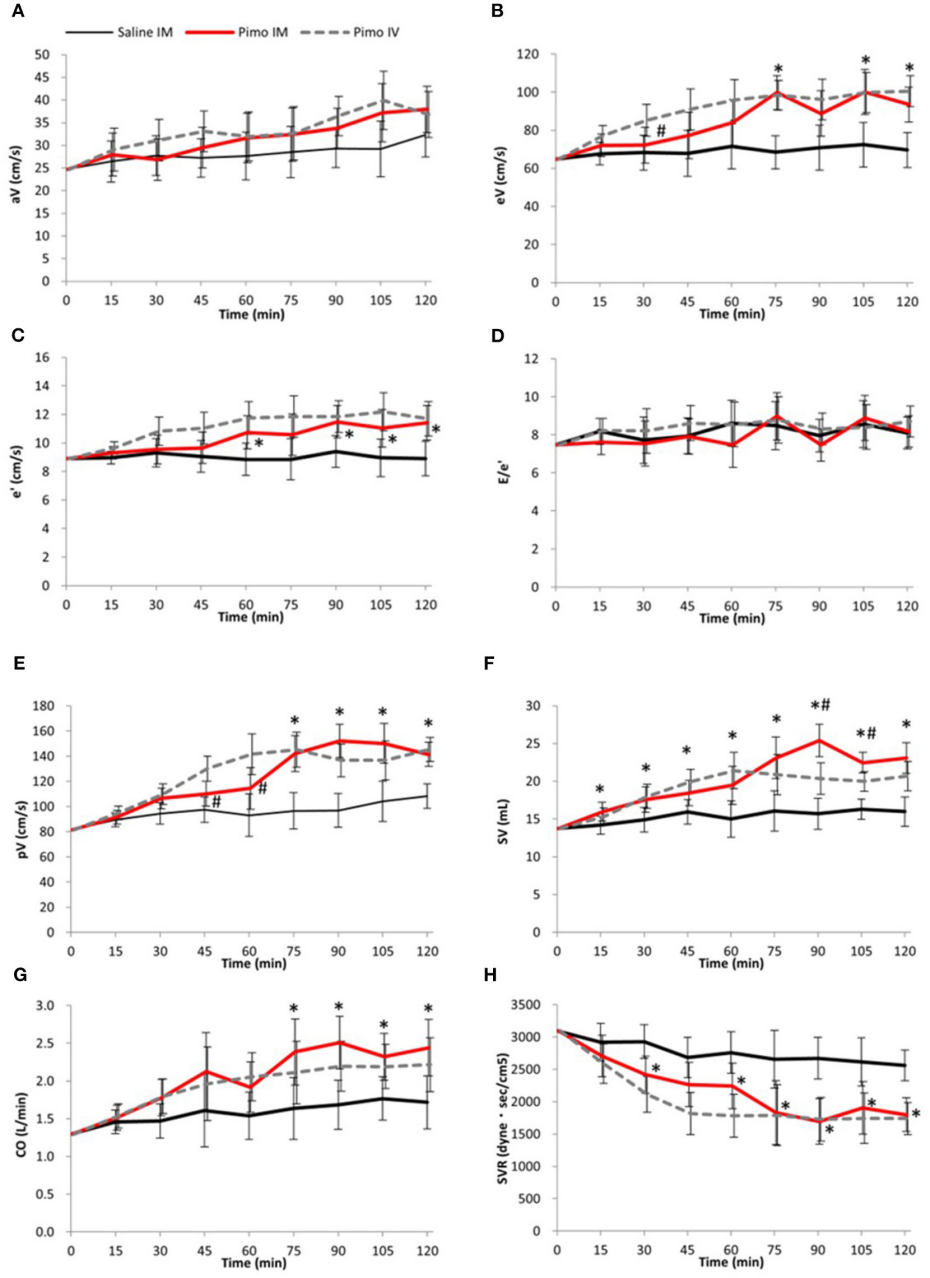
Data are presented as mean ± 95% confidence interval (error bar). Black line: Saline IM; Red line: Pimobendan IM (Pimo IM); Gray dot line: Pimobendan IV (Pimo IV). (*) fitted to indicate the significant difference between Pimo IM and Saline IM, while (#) fitted to compare Pimo IM vs. Pimo IV [for the repeated measures ANCOVA (model: time, group, and time*group)]; *P* < 0.05. **(A)** Peak atrial filling velocity (aV, cm/s), **(B)** Early diastolic left ventricular filling velocity (eV, cm/s), **(C)** Peak early diastolic velocity of the mitral annulus (e′, cm/s), **(D)** Early diastolic left ventricular filling velocity/Peak early diastolic velocity of the mitral annulus ratio (E/e′), **(E)** Peak velocity (pV, cm/s), **(F)** Stroke volume (SV, mL), **(G)** Cardiac output (CO, L/min), **(H)** Systemic vascular resistance (SVR, dyne·s/cm^5^).

In the Pimo IM group, a significant increase in pV was observed at 75 min [45.45 (CI: 24.69 - 66.21), *P* < 0.001; Figure 2E], for SV at 15 min [1.77 (0.02 - 3.5), *P* = 0.048; Figure 2F], and for CO at 75 min [0.75 (CI: 0.13 - 1.37), *P* = 0.022; Figure 2G] after administrations compared with the saline IM.

No significant difference between groups was observed in late mitral velocity (aV) at all measurement points; however, early mitral velocity (eV) was significantly increased at 75 min [31.30 (CI: 17.65 - 44.94), *P* < 0.001], 105 min [27.76 (CI: 9.49 - 46.04), *P* = 0.006], and 120 min [23.88 (CI: 9.70 - 38.06), *P* = 0.003] after Pimo IM administration compared with saline IM (Figures 2A,B).

In the Pimo IM group, mitral annulus velocity (e′) was significantly reduced at 60 min [1.89 (0.27 - 3.51), *P* = 0.025] and 90 min [2.08 (0.50 - 3.66), *P* = 0.014] after administration compared with the placebo (Figure 2C). There was no significant difference in E/e′ ratio between Pimo IM and saline IM at all measurement points (Figure 2D). Finally, SVR showed a significant decrease in the Pimo IM group after 30 min [−514.2 (CI: −917.6 - −110.8), *P* = 0.016] and 60 min [−515.7 (CI: −1003.6 - −27.9), *P* = 0.040] of administration (Figure 2H).

### Comparison between the two routes of pimobendan administration

In the Pimo IM and IV groups, significant changes were observed in eV 30 min after administration [difference between groups (“Pimo IM,” “Pimo IV”): Time 30: −12.98 (CI: −25.57 - −0.39), *P* = 0.044; Figure 2B] and in pV at 45 min [−19.96 (CI: −33.88 - −6.05), *P* = 0.008] and 60 min [−27.07 (CI: −50.07 - −4.06), *P* = 0.024] after administration (Figure 2E), with a significant increase in the Pimo IV group. On the other hand, SV was significantly increased in Pimo IM at 90 min [5.07 (CI: 2.02 - 8.12), *P* = 0.003] and 105 min [2.47 (CI: 0.53 - 4.40), *P* = 0.016] compared with Pimo IV (Figure 2F). In addition, there was no significant difference in BP, HR, aV, SVR, CO, e′, and E/e′ between IM and IV routes of pimobendan injection (Figures 1A–D, 2A,C,D,G,H).

## Discussion

Pimobendan is a drug known to enhance the systolic force and vasodilator effects, and so, is defined as an inodilator (11). In the present study, we measured parameters that represent systolic contractile and diastolic relaxing functions of LV myocardium and BP to verify whether IM injectable pimobendan works safely and effectively in healthy dogs. Our results showed that the two routes of pimobendan administration affected the LV myocardial diastolic and systolic functions by increasing eV of the mitral inflow and pV values of LV outflow (relaxation) and increasing SV and CO (positive inotropy), along with the superiority of pimobendan IM over IV. Also, IM pimobendan produced vasodilator effects due to decreasing SVR and diastolic BP within 120 min of a single dose in healthy dogs.

Pimobendan, a benzimidazole-pyridazinone derivative, is a non-sympathomimetic, and non-glycoside inotropic agent that presents vasodilator effects by inhibiting PDE-III, a regulator of cardiac and vascular smooth muscle contractility (11). Additionally, pimobendan has a Ca^+2^-sensitizing effect and directly increases the affinity of the regulatory site on cardiac troponin C for Ca^+2^, giving rise to a positive inotropic activity (12–14). Pimobendan increases CO and decreases SVR (inodilation) in a dose-dependent manner. In the current study, the observed increases in CO and decreases in SVR following IM pimobendan administration were consistent with a previous study (24). These results show that the inodilator effect of pimobendan is maintained after IM injection.

Rapid fluctuations in the HR and BP may be a high risk for acute heart failure patients who are already in shock with hypotension. In the present study, a significant increase in HR was observed in Pimo IM and saline IM groups, but the amount of change from the baseline in those two groups was almost the same (~10 beats). This finding indicated that the increase in HR was related to factors other than the effect of pimobendan, such as the effect of long-term anesthesia. Isoflurane was maintained at ±0.1% during the experiment, ETCO2 was 35–45 mmHg, and body temperature was maintained at 37.0–37.5°C. However, the T1/2 of propofol used in the anesthetic preadministration was 4.2 ± 2.2 min (33) and the T1/2 of butorphanol was 1.3–3.6 h. It is possible that the attenuation of these effects led to a decrease in anesthetic depth, which in turn led to an increase in heart rate. Therefore, IM (and IV) administrations of pimobendan (0.15 mg/kg) were considered not to affect the HR, SBP, or MBP. This is in agreement with a previous study in which pimobendan was administered intravenously (23). On the other hand, some studies have reported an increase in HR and a decrease in systolic LV pressure (34, 35). Other studies also stated that the increase in HR and decrease in BP after pimobendan administration is dose-dependent (36, 37). The differential effect of pimobendan on HR and BP may be due to the differences in the dose of pimobendan among the studies. When used at high doses as in later studies, the effect of pimobendan on HR and BP will be more than the net effect observed in the present study.

Reducing the afterload of the LV is an important target during the treatment of acute heart failure. The afterload is mainly due to the resistance applied to the LV wall and the resistance of the arterial system. The resistance applied to the LV wall is defined by SBP, while the resistance of the arterial system is defined by SVR (2). In the present study, although no significant change was observed in SBP, a significant decrease was observed in the SVR, indicating that IM pimobendan leads to vasodilation. In addition, the vasodilator effect was rapidly observed (after 15 min) and was maximized 90 min after administration, the same as observed *via* the IV route. Pimobendan is given primarily to increase CO (11). Hypotension is frequently observed in cases of cardiogenic shock in which an immediate increase in CO should be considered to maintain effective circulation (25, 29). Our findings suggested that IM pimobendan is not a hypotensive drug, and therefore will not worsen the blood pressure in case of hypotension.

Active relaxation of the LV myocardium occurs when the LV pressure drops below LAP, the pressure difference becomes the driving pressure and the mitral valve opens, causing blood inflow from the left atrium to the LV; this is eV (38). In the present study, pimobendane IM administration resulted in a significant increase in eV. The potential reasons for eV increase are the active relaxation of the LV or increased LAP (39). Previous studies have reported that pimobendan significantly reduces the trans LAP (15) and LV end-diastolic pressure (23). Moreover, the present study showed a significant increase in e′; e′ represents myocardial movement during diastole (40). Therefore, this result indicated that the IM pimobendan was effective regarding cardiotonic action. In addition, there was no difference in E/e′ in the present study. Previous studies reported a significant correlation between E/e′ and mean LAP (40, 41). There was no significant change in LAP in dogs treated with pimobendan in the current study. Nevertheless, Ohte et al. (35) found a significant decrease in LAP when pimobendan was administered at a high dose (0.25 mg/kg). Lowering LAP is necessary in heart failure treatment. However, the low dose of pimobendan (0.15 mg/kg) in our study did not decrease LAP. Therefore, the absence of reduced LAP in our study could be attributed to the difference in pimobendan doses used in this study compared with other studies (36).

Cardiac output is a result of the synergistic action of cardiotonic activity, preload, and afterload (2). The results of the present study demonstrated a rapid and marked increase in CO after IM administration of pimobendan, most probably due to either increased cardiotonic action, increased preload, decreased afterload, or the synergistic effect (11, 16). The present study did not measure factors increasing the preload. As described above, the results of SVR and e′ values were thought to be compatible with a reduction in the afterload and an increase of cardiotonic action, respectively. Therefore, it was considered that the increase in the CO in the present study was due to the enhancement of cardiotonic action and the reduction of afterload. Cardiac output is proportional to the initial length of myocardial fibers as defined by Frank-Starling's law (42). This means that CO depends on the relaxation of the left ventricular myocardium and its ability to contract. The results of pimobendan administration obtained in this study indicated that pimobendan contributes to increased CO by enhancing myocardial relaxation, thereby promoting an increase in blood volume during left ventricular relaxation and enhancing myocardial contractile capacity (35, 43, 44). In addition, the enhanced vasodilatory effects decreased peripheral vascular resistance and reduced afterload, which was thought to be attributable to the increase in CO (35, 43, 45).

In this study, the hemodynamic effect of IM and IV pimobendan has been comparatively described. Significance difference was only observed in eV, SV, and pV while other parameters (BP, HR, SVR, CO, e′, and E/e′) were not significantly changed. However, in comparison with the baseline, while pV, SV, and CO showed statistically significant increases after pimobendan injection *via* the two routes, SVR decreased significantly after 15 min in both groups, indicating that the two routes are approximately expressing the same hemodynamic effect with extended improve in SV after IM administration. Our results suggested that both IM and IV administration of pimobendan nearly induce the same hemodynamic effect and multiple routes of the drug administration will be more beneficial for life-saving therapies, especially in urgent conditions.

Moreover, there were significant differences in eV and e′ values between IM and IV routes of pimobendan administration. Generally, eV and e′ are measured to assess LV myocardial relaxation using echocardiography (38, 40). The IM administration of pimobendan caused a significant increase in the eV after Time 45 and e′ after Time 60, whereas its IV administration caused a significant increase in both eV and e′ after 15 min. From these results, it was speculated that the IM injection of pimobendan might have a milder effect on the LV myocardial relaxation than its IV administration. This was thought to be due to the fact that when pimobendan is administered intramuscularly, it takes time for the drug to move from the intramuscular to the bloodstream (26, 27). In the present study, this difference in time was thought to account for the different effects of IV and IM administration. In this regard, it can be considered that IM is another choice in acute heart failure patients who are already in shock with collapsed peripheral vessels. Because of the availability of oral, IV and IM routes of pimobendan administration, it will be easier for the practitioner to choose a suitable route of its administration depending on the condition of each case particularly in countries where injectable pimobendan solution is available. However, injectable pimobendan may be extended soon to include many countries (17, 24). In the future, further research is needed to accurately evaluate how the effects of cardiotonic action, vasodilatory action, and LV myocardial relaxation action change depending on different methods of pimobendan administration in clinical cases.

### Clinical implications

Taken together, our results suggested that the IM route of pimobendan administration is an effective method to provide cardiotonic action, vasodilator effect, and LV myocardial relaxation. At present, IV administrations of cardiotonic drugs have been preferred, especially in the emergency treatment of heart failure in human and veterinary medicine. However, IM pimobendan might be another simple and safe route for cases in which the heart failure is complicated by other factors such as hemoptysis, and when the condition is expected to worsen due to stress—caused, for example, by retention to secure a venous route—and in cases of cardiogenic shock associated with difficulty in securing a venous route. In the current study, anesthesia resulted in some hemodynamic changes as previously reported (46–50), however, our results provided clear evidence for the usefulness of IM pimobendan the same as observed by IV pimobendane to enhance the heart function in healthy dogs.

### Limitations

The current study has been conducted on healthy dogs. Hemodynamic properties of pimobendan are different in animals with heart disease compared to healthy dogs and the sensitivity by target site may vary depending on the degree of acidemia and circulatory failure in dogs with CHF. Here we have to say that various routes of injectable pimobendan administration are still under research consideration using animal models or healthy dogs and further clinical studies should be performed (17, 21–23).

We did not measure the plasma concentration of pimobendan and its active metabolite following IM injection. In our protocol, we measured the hemodynamic parameters every 15 minutes. This requires continuous blood sampling at short intervals which may impact the hemodynamics during the study. In addition, it has been reported that pimobendan has altered pharmacokinetics in plasma, whole blood, and intracellularly in dogs (17).

Besides, the effects of pimobendan administration on CO and vasodilation were measured by echocardiography. Despite cardiac catheterization being more accurate than echocardiography in these measurements, cardiac catheterization is more invasive and unsuitable for repeated measurements in each dog (51–53). Moreover, we elected to conduct the study under anesthesia to avoid any fluctuation in cardiac measurements during the handling of animals as previously described (22, 23). However, the isoflurane used in the present study has a dose-dependent antihypertensive effect associated with vasodilation and negative inotropic effects as previously reported (46–49); additionally, isoflurane has been reported to increase the HR and LAP and decrease myocardial contractility (50). This can diminish the effect of pimobendan even though the hemodynamic effect of injectable pimobendan in our study was clear.

## Conclusion

The present study showed that intramuscular pimobendan changes cardiovascular functions by enhancing LV myocardial relaxation and LV contractility, producing vasodilation within a short time after its administration in healthy dogs approximately in the same way as the IV route. However, further clinical studies should be performed to explore the usefulness of an intramuscular administration of Pimobendan and whether it is an effective route of administration in case of emergency and when the IV route cannot be applied.

## Data availability statement

The raw data supporting the conclusions of this article will be made available by the authors, without undue reservation.

## Ethics statement

The present study was carried out according to the standards set by the Tokyo University of Agriculture and Technology (TUAT) and the guide on the use of laboratory animals. All experimental procedures were approved by the TUAT Animal Care and Use Committee (Approval No. R03-180).

## Author contributions

ME and RT conceived and designed the experiment. ME, ASM, KS, SK, KK, and AY methodology and data collection. ME, SG, and KS animal preparation and anesthesia. ZY, SG, and KS data validation. ME and ASM statistical analysis, manuscript drafting, and writing the final manuscript. All authors have read and agreed to the published version of the manuscript.

## Conflict of interest

The authors declare that the research was conducted in the absence of any commercial or financial relationships that could be construed as a potential conflict of interest.

The handling editor HS declared a past co-authorship with the authors ASM and RT.

## Publisher's note

All claims expressed in this article are solely those of the authors and do not necessarily represent those of their affiliated organizations, or those of the publisher, the editors and the reviewers. Any product that may be evaluated in this article, or claim that may be made by its manufacturer, is not guaranteed or endorsed by the publisher.

## Abbreviations

aV: The left ventricular inflow waveforms of the atrial systolic wave
BP: Blood pressure
CHF: Congestive heart failure
CO: Cardiac output
DBP: Diastolic blood pressure
DCM: Dilated cardiomyopathy
E/e′: early mitral inflow to the diastolic early mitral ring velocity
eV: The left ventricular inflow waveforms of the diastolic early wave
HR: Heart rate
LAP: Left atrial pressure
LV: Left ventricular
LVEDP: The left ventricular end-diastolic pressure
MAP: Mean blood pressure
MVD: Mitral valve disease
PaCO_2_: Expiratory terminal carbon dioxide partial pressure
PDE-III: Phosphodiesterase III
Pimo IM: Intramuscular administration of pimobendan
Pimo IV: Intravenous administration of pimobendan
pV: Peak velocity
SBP: Systolic blood pressure
SpO_2_: Saturation of percutaneous oxygen
SV: Stroke volume
SVR: Systemic vascular resistance
VTI: Velocity-time integral.

## References

[1] Cheng CJ Mandour AS Yoshida T Watari T Tanaka R Matsuura K. Changes in renin-angiotensin-aldosterone system during cardiac remodeling after mitral valvuloplasty in dogs. J Vet Intern Med. (2022) 36:397–405. 10.1111/jvim.16346 34994485 PMC8965262

[2] Tilley LP. Manual of Canine and Feline Cardiology. West Sussex: Elsevier Health Sciences (2008).

[3] Boswood A Häggström J Gordon SG Wess G Stepien RL Oyama MA. Effect of pimobendan in dogs with preclinical myxomatous mitral valve disease and cardiomegaly: the EPIC study-a randomized clinical trial. J Vet Intern Med. (2016) 30:1765–79. 10.1111/jvim.14586 27678080 PMC5115200

[4] Häggström J Boswood A O'Grady M Jöns O Smith S Swift S. Longitudinal analysis of quality of life, clinical, radiographic, echocardiographic, and laboratory variables in dogs with myxomatous mitral valve disease receiving pimobendan or benazepril: the QUEST study. J Vet Intern Med. (2013) 27:1441–51. 10.1111/jvim.12181 24010489

[5] Häggström J Lord PF Höglund K Ljungvall I Jöns O Kvart C. Short-term hemodynamic and neuroendocrine effects of pimobendan and benazapril in dogs with myxomatous mitral valve disease and congestive heart failure. J Vet Intern Med. (2013) 27:1452–62. 10.1111/jvim.12217 24128373

[6] O'Grady MR Minors SL O'Sullivan ML Horne R. Effect of pimobendan on case fatality rate in Doberman Pinschers with congestive heart failure caused by dilated cardiomyopathy. J Vet Intern Med. (2008) 22:897–904. 10.1111/j.1939-1676.2008.0116.x 18537880

[7] Keene BW Atkins CE Bonagura JD Fox PR Häggström J Fuentes VL. ACVIM consensus guidelines for the diagnosis and treatment of myxomatous mitral valve disease in dogs. J Vet Intern Med. (2019) 33:1127–40. 10.1111/jvim.15488 30974015 PMC6524084

[8] Iwanuk N Wall L Nolte I Raue J Rumstedt K Pilgram A. Effect of pimobendan on physical fitness, lactate and echocardiographic parameters in dogs with preclinical mitral valve disease without cardiomegaly. PLoS ONE. (2019) 14:e0223164. 10.1371/journal.pone.0223164 31581204 PMC6776412

[9] Böhm M Morano I Pieske B Rüegg JC Wankerl M Zimmermann R. Contribution of cAMP-phosphodiesterase inhibition and sensitization of the contractile proteins for calcium to the inotropic effect of pimobendan in the failing human myocardium. Circ Res. (1991) 68:689–701. 10.1161/01.RES.68.3.689 1660359

[10] Fujino K Sperelakis N Solaro RJ. Sensitization of dog and guinea pig heart myofilaments to Ca2+ activation and the inotropic effect of pimobendan: comparison with milrinone. Circ Res. (1988) 63:911–22. 10.1161/01.RES.63.5.911 2846200

[11] Fitton A Brogden RN. Pimobendan. A review of its pharmacology and therapeutic potential in congestive heart failure. Drugs Aging. (1994) 4:417–41. 10.2165/00002512-199404050-00007 8043944

[12] Brunkhorst D v der Leyen H Meyer W Nigbur R Schmidt-Schumacher C Scholz H. Relation of positive inotropic and chronotropic effects of pimobendan, UD-CG 212 Cl, milrinone and other phosphodiesterase inhibitors to phosphodiesterase III inhibition in guinea-pig heart. Naunyn-Schmiedeberg's Arch Pharmacol. (1989) 339:575–83. 10.1007/BF00167264 2549430

[13] Duncker DJ Hartog JM Levinsky L Verdouw PD. Systemic haemodynamic actions of pimobendan (UD-CG 115 BS) and its O-demethylmetabolite UD-CG 212 Cl in the conscious pig. Br J Pharmacol. (1987) 91:609–15. 10.1111/j.1476-5381.1987.tb11254.x 3607369 PMC1853547

[14] Lee JA Ruegg JC Allen DG. Effects of pimobendan, a novel inotropic agent, on intracellular calcium and tension in isolated ferret ventricular muscle. Clin Sci. (1989) 76:609–18. 10.1042/cs0760609 2544344

[15] Suzuki S Fukushima R Ishikawa T Hamabe L Aytemiz D Huai-Che H. The effect of pimobendan on left atrial pressure in dogs with mitral valve regurgitation. J Vet Intern Med. (2011) 25:1328–33. 10.1111/j.1939-1676.2011.00800.x 22092624

[16] Boyle KL Leech E. A review of the pharmacology and clinical uses of pimobendan. J Vet Emerg Crit Care. (2012) 22:398–408. 10.1111/j.1476-4431.2012.00768.x 22928748

[17] Bell ET Devi JL Chiu S Zahra P Whittem T. The pharmacokinetics of pimobendan enantiomers after oral and intravenous administration of racemate pimobendan formulations in healthy dogs. J Vet Pharmacol Ther. (2016) 39:54–61. 10.1111/jvp.12235 25989021

[18] Her J Kuo KW Winter RL Cruz-Espindola C Bacek LM Boothe DM. Pharmacokinetics of pimobendan and its metabolite O-desmethyl-pimobendan following rectal administration to healthy dogs. Front Vet Sci. (2020) 7:423. 10.3389/fvets.2020.00423 32851013 PMC7417621

[19] Pichayapaiboon P Tantisuwat L Boonpala P Saengklub N Boonyarattanasoonthorn T Khemawoot P. Pharmacodynamics and pharmacokinetics of injectable pimobendan and its metabolite, O-desmethyl-pimobendan, in healthy dogs. Front Vet Sci. (2021) 8:656902. 10.3389/fvets.2021.656902 34490386 PMC8417876

[20] Yata M McLachlan AJ Foster DJ Page SW Beijerink NJ. Pharmacokinetics and cardiovascular effects following a single oral administration of a nonaqueous pimobendan solution in healthy dogs. J Vet Pharmacol Ther. (2016) 39:45–53. 10.1111/jvp.12243 25997373

[21] Morita T Nakamura K Osuga T Kawamoto S Miki S Sasaoka K. Acute effects of intravenous pimobendan administration in dog models of chronic precapillary pulmonary hypertension. J Vet Cardiol. (2020) 32:16–27. 10.1016/j.jvc.2020.09.003 33080489

[22] Miki S Nakamura K Morita T Osuga T Kawamoto S Sasaki N. Effects of intravenous administration of pimobendan on hemodynamic indices and indices of left atrial longitudinal strain derived from speckle-tracking echocardiography in healthy dogs. Am J Vet Res. (2021) 82:795–801. 10.2460/ajvr.82.10.795 34554874

[23] Hori Y Taira H Nakajima Y Ishikawa Y Yumoto Y Maekawa Y. Inotropic effects of a single intravenous recommended dose of pimobendan in healthy dogs. J Vet Med Sci. (2019) 81:22–5. 10.1292/jvms.18-0185 30404952 PMC6361644

[24] Itami T Hanazono K Makita K Yamashita K. Cardiovascular effects of intravenous pimobendan in dogs with acute respiratory acidosis. J Vet Emerg Crit Care. (2022) 32:341–9. 10.1111/vec.13178 35080109

[25] Silverstein DC Kleiner J Drobatz KJ. Effectiveness of intravenous fluid resuscitation in the emergency room for treatment of hypotension in dogs: 35 cases (2000–2010). J Vet Emerg Crit Care. (2012) 22:666–73. 10.1111/j.1476-4431.2012.00822.x 23216841

[26] Rebuelto M Albarellos G Ambros L Kreil V Montoya L Bonafine R. Pharmacokinetics of ceftriaxone administered by the intravenous, intramuscular or subcutaneous routes to dogs. J Vet Pharmacol Ther. (2002) 25:73–6. 10.1046/j.1365-2885.2002.00389.x 11874531

[27] Schwartz M Muñana KR Nettifee-Osborne JA Messenger KM Papich MG. The pharmacokinetics of midazolam after intravenous, intramuscular, and rectal administration in healthy dogs. J Vet Pharmacol Ther. (2013) 36:471–7. 10.1111/jvp.12032 23256899

[28] Yoshida T Mandour AS Matsuura K Shimada K El-Husseiny HM Hamabe L. Changes in the pulmonary artery wave reflection in dogs with experimentally-induced acute pulmonary embolism and the effect of vasodilator. Animals Open Access J MDPI. (2021) 11:977. 10.3390/ani11071977 34359104 PMC8300366

[29] Yoshida T Matsuura K Mandour AS Aboshi Y Yamada S Yotsuida H. Hemodynamic effects of protamine infusion in dogs with myxomatous mitral valve disease undergoing mitral valvuloplasty. Vet Sci. (2022) 9:178. 10.3390/vetsci9040178 35448675 PMC9031179

[30] Boon JA. Veterinary Echocardiography. West Sussex: John Wiley & Sons. (2011).

[31] Haskins S Pascoe PJ Ilkiw JE Fudge J Hopper K Aldrich J. Reference cardiopulmonary values in normal dogs. Comp Med. (2005) 55:156–61. 15884778

[32] Fukushima R Kawaguchi T Yamada S Yoshimura A Hirao D Oomori T. Effects of cilostazol on the heart rate in healthy dogs. J Vet Med Sci. (2018) 80:1707–15. 10.1292/jvms.18-0240 30249936 PMC6261822

[33] Cockshott ID Douglas EJ Plummer GF Simons PJ. The pharmacokinetics of propofol in laboratory animals. Xenobiotica. (1992) 22:369–75. 10.3109/00498259209046648 1496826

[34] Asanoi H Ishizaka S Kameyama T Ishise H Sasayama S. Disparate inotropic and lusitropic responses to pimobendan in conscious dogs with tachycardia-induced heart failure. J Cardiovasc Pharmacol. (1994) 23:268–74. 10.1097/00005344-199402000-00014 7511757

[35] Ohte N Cheng CP Suzuki M Little WC. The cardiac effects of pimobendan (but not amrinone) are preserved at rest and during exercise in conscious dogs with pacing-induced heart failure. J Pharmacol Exp Ther. (1997) 282:23–31. 9223536

[36] Pagel PS Hettrick DA Warltier DC. Influence of levosimendan, pimobendan, and milrinone on the regional distribution of cardiac output in anaesthetized dogs. Br J Pharmacol. (1996) 119:609–15. 10.1111/j.1476-5381.1996.tb15716.x 8894186 PMC1915696

[37] van Meel JC Diederen W. Hemodynamic profile of the cardiotonic agent pimobendan. J Cardiovasc Pharmacol. (1989) 14(Suppl. 2):S1–6. 10.1097/00005344-198906142-00002 2478784

[38] Nagueh SF. Left Ventricular diastolic function: understanding pathophysiology, diagnosis, and prognosis with echocardiography. JACC Cardiovasc Imag. (2020) 13:228–44. 10.1016/j.jcmg.2018.10.038 30982669

[39] Masuyama T Popp RL. Doppler evaluation of left ventricular filling in congestive heart failure. Eur Heart J. (1997) 18:1548–56. 10.1093/oxfordjournals.eurheartj.a015134 9347265

[40] Nagueh SF Middleton KJ Kopelen HA Zoghbi WA Quiñones MA. Doppler tissue imaging: a noninvasive technique for evaluation of left ventricular relaxation and estimation of filling pressures. J Am Coll Cardiol. (1997) 30:1527–33. 10.1016/S0735-1097(97)00344-6 9362412

[41] Ommen SR Nishimura RA Appleton CP Miller FA Oh JK Redfield MM. Clinical utility of Doppler echocardiography and tissue doppler imaging in the estimation of left ventricular filling pressures: a comparative simultaneous Doppler-catheterization study. Circulation. (2000) 102:1788–94. 10.1161/01.CIR.102.15.1788 11023933

[42] Sunagawa K. The pressure-volume relationship of the heart: past, present and future. Ann Int Conf IEEE Eng Med Biol Soc. (2010) 2010:3554–5. 10.1109/IEMBS.2010.5627485 21096827

[43] Hall J Guyton A. Cardiac muscle; the heart as a pump and function of the heart valves. In: Guyton and Hall Textbook of Medical Physiology. New York, NY: Elsevier Inc (2016). p. 12.

[44] He KL Dickstein M Sabbah HN Yi GH Gu A Maurer M. Mechanisms of heart failure with well preserved ejection fraction in dogs following limited coronary microembolization. Cardiovasc Res. (2004) 64:72–83. 10.1016/j.cardiores.2004.06.007 15364615

[45] Isoyama S Maruyama Y Koiwa Y Ishide N Kitaoka S Tamaki K. Experimental study of afterload-reducing therapy: the effects of the reduction of systemic vascular resistance on cardiac output, aortic pressure and coronary circulation in isolated, ejecting canine hearts. Circulation. (1981) 64:490–9. 10.1161/01.CIR.64.3.490 7261281

[46] Boban M Stowe DF Buljubasic N Kampine JP Bosnjak ZJ. Direct comparative effects of isoflurane and desflurane in isolated guinea pig hearts. Anesthesiology. (1992) 76:775–80. 10.1097/00000542-199205000-00016 1575346

[47] Hettrick DA Pagel PS Warltier DC. Desflurane, sevoflurane, and isoflurane impair canine left ventricular-arterial coupling and mechanical efficiency. Anesthesiology. (1996) 85:403–13. 10.1097/00000542-199608000-00023 8712457

[48] Rusy BF Komai H. Anesthetic depression of myocardial contractility: a review of possible mechanisms. Anesthesiology. (1987) 67:745–66. 10.1097/00000542-198711000-00020 3314598

[49] Swanson CR Muir WW III. Simultaneous evaluation of left ventricular end-systolic pressure-volume ratio and time constant of isovolumic pressure decline in dogs exposed to equivalent MAC halothane and isoflurane *Anesthesiology*. (1988) 68:764–70. 10.1097/00000542-198805000-00015 3369716

[50] Seifen E Seifen AB Kennedy RH Bushman GA Loss GE Jr Williams TG. Comparison of cardiac effects of enflurane, isoflurane, and halothane in the dog heart-lung preparation. J Cardiothor Anesth. (1987) 1:543–53. 10.1016/0888-6296(87)90041-X 17165353

[51] Lopes PC Sousa MG Camacho AA Carareto R Nishimori CT Santos PS. Comparison between two methods for cardiac output measurement in propofol-anesthetized dogs: thermodilution and Doppler. Vet Anaesth Analg. (2010) 37:401–8. 10.1111/j.1467-2995.2010.00552.x 20712606

[52] Matsuura K Shiraishi K Mandour AS Sato K Shimada K Goya S. The utility of intraventricular pressure gradient for early detection of chemotherapy-induced subclinical cardiac dysfunction in dogs. Animals Open Access J MDPI. (2021) 11:1122. 10.3390/ani11041122 33919889 PMC8070943

[53] Miki H Mandour AS Goya S Hamabe L Matsuura K Yoshida T. Color M-mode echocardiography for non-invasive assessment of the intraventricular pressure in dogs before and after ductus arteriosus occlusion: a retrospective study. Front Vet Sci. (2022) 9:908829. 10.3389/fvets.2022.908829 35903130 PMC9315367

